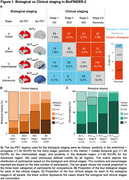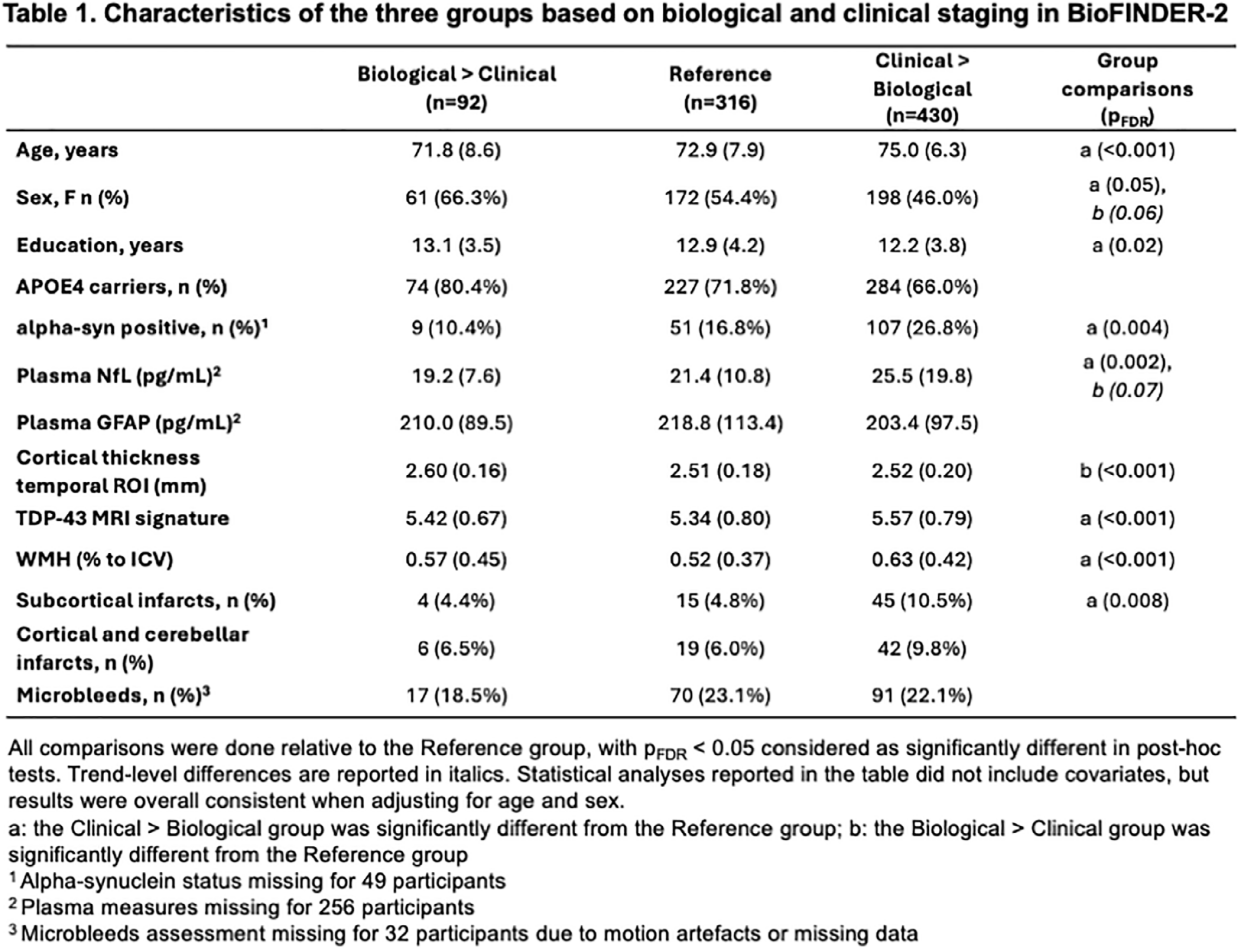# Evaluation of the revised criteria for biological and clinical staging of Alzheimer's disease

**DOI:** 10.1002/alz70856_098208

**Published:** 2025-12-24

**Authors:** Alexa Pichet Binette, Ruben Smith, Gemma Salvadó, Pontus Tideman, Isabelle Glans, Danielle van Westen, Colin Groot, Rik Ossenkoppele, Erik Stomrud, Piero Parchi, Henrik Zetterberg, Kaj Blennow, Niklas Mattsson‐Carlgren, Shorena Janelidze, Sebastian Palmqvist, Oskar Hansson

**Affiliations:** ^1^ Clinical Memory Research Unit, Lund University, Lund, Sweden; ^2^ Université de Montréal, Montreal, QC, Canada; ^3^ Centre de Recherche de l’Institut Universitaire de Gériatrie de Montréal, Montreal, QC, Canada; ^4^ Clinical Memory Research Unit, Lund University, Malmö, Skåne, Sweden; ^5^ Memory Clinic, Skåne University Hospital, Malmö, Skåne, Sweden; ^6^ Barcelonaβeta Brain Research Center (BBRC), Pasqual Maragall Foundation, Barcelona, Spain; ^7^ Diagnostic Radiology, Institute for Clinical Sciences Lund, Lund University, Lund, Sweden; ^8^ Imaging and Function, Skåne University Hospital, Lund, Sweden; ^9^ Alzheimer Center Amsterdam, Neurology, Vrije Universiteit Amsterdam, Amsterdam UMC location VUmc, Amsterdam, Netherlands; ^10^ Clinical Memory Research Unit, Department of Clinical Sciences Malmö, Faculty of Medicine, Lund University, Lund, Sweden; ^11^ IRCCS Istituto delle Scienze Neurologiche di Bologna, Bologna, Italy; ^12^ University of Bologna, Bologna, Italy; ^13^ Hong Kong Center for Neurodegenerative Diseases, Hong Kong, Science Park, China; ^14^ Wisconsin Alzheimer's Disease Research Center, University of Wisconsin‐Madison, School of Medicine and Public Health, Madison, WI, USA; ^15^ Institute of Neuroscience and Physiology, Sahlgrenska Academy at the University of Gothenburg, Gothenburg, Sweden; ^16^ Clinical Neurochemistry Laboratory, Sahlgrenska University Hospital, Gothenburg, Sweden; ^17^ UCL Institute of Neurology, London, United Kingdom; ^18^ Paris Brain Institute, ICM, Pitié‐Salpêtrière Hospital, Sorbonne University, Paris, France; ^19^ Clinical Neurochemistry Laboratory, Sahlgrenska University Hospital, Mölndal, Sweden; ^20^ Institute of Neuroscienace and Physiology, University of Gothenburg, Mölndal, Västra Götaland, Sweden; ^21^ Department of Neurology, Skåne University Hospital, Lund, Sweden

## Abstract

**Background:**

In the recent update of the Alzheimer's disease (AD) criteria, tau‐PET was introduced as a core biomarker, and its spatial extent was incorporated into the revised biological stages of the disease. Our objectives were to (1) implement the new criteria in a large research cohort and (2) compare individuals who have discrepant biological and clinical stages to those who have congruent stages in terms of co‐pathologies and demographics.

**Method:**

We included 838 amyloid‐b‐positive participants from the BioFINDER‐2 cohort who had undergone tau‐PET ([^18^F]RO948). We classified them on clinical staging (cognitively normal to dementia) and biological staging (early to advanced) (Figure 1A). We then compared individuals with congruent biological and clinical stage ("Reference" group) to those with more advanced clinical impairment than biological stage ("Clinical > Biological") and to those with the opposite pattern ("Biological > Clinical") on demographics, measures of neurodegeneration (cortical thickness, TDP‐43 MRI signature, NfL), alpha‐synuclein CSF status, GFAP, white matter lesions, infarcts and microbleeds.

**Result:**

37.7% of the sample were in the Reference group (grey squares), 51.3% were in the Clinical > Biological group (red squares) and 11.0% were in the opposite group, Biological > Clinical (blue squares) (Figure 1). The main differences (all results in Table 1) were between the Reference group and the Clinical > Biological group: the latter participants were older, included more men, were more often positive for alpha‐synuclein pathology, had higher NfL levels, greater TDP‐43 like atrophy and higher burden of cerebral small vessel disease lesions (all p_FDR_<0.05). The only difference observed between the Biological > Clinical and the Reference group was that the former had less neurodegeneration (thicker cortex, p_FDR_<0.01). Results were also consistent when analyses were adjusted for age and sex, or when further splitting the sample in five subgroups instead of three.

**Conclusion:**

In this study we validated the new AD staging criteria and found that co‐pathologies play an important role in symptom severity in individuals harboring less tau tangle pathology than expected for their clinical impairment. These results highlight the importance of measuring non‐AD biomarkers in patients with worse cognitive impairment than expected based on their biological stage, which could impact clinical diagnosis and prognosis.